# Guideline summary: assessment, diagnosis, care and support for people with dementia and their carers [Scottish Intercollegiate Guidelines Network SIGN Guideline 168]

**DOI:** 10.1093/ageing/afae147

**Published:** 2024-07-06

**Authors:** Jennifer Kirsty Burton, Roy L Soiza, Terence J Quinn, On behalf of the SIGN 168 guideline development group

**Affiliations:** Academic Geriatric Medicine, School of Cardiovascular and Metabolic Health, College of Medicine, Veterinary and Life Sciences, University of Glasgow, Glasgow G31 2ER, UK; Ageing Clinical and Experimental Research Group, Institute of Applied Health Sciences, University of Aberdeen, Aberdeen AB25 2ZD, UK; Academic Geriatric Medicine, School of Cardiovascular and Metabolic Health, College of Medicine, Veterinary and Life Sciences, University of Glasgow, Glasgow G31 2ER, UK

**Keywords:** older people, Alzheimer’s, dementia, guideline

## Abstract

The Scottish Intercollegiate Guidelines Network (SIGN) have recently published their guideline SIGN168 on ‘Assessment, Diagnosis, Care, and Support for People with Dementia and their Carers’. The guideline makes evidence-based recommendations for best practice in the assessment, care and support of adults living with dementia. Topics featured in this guideline are limited to those prioritised by stakeholders, especially people with lived and living experience, and those not well covered under pre-existing guidance. We summarise the guideline recommendations related to identification and diagnosis of dementia, investigative procedures, postdiagnostic support living with dementia, including non-pharmacological approaches for distressed behaviours, using technology to support people with dementia, grief and dementia and changing needs of people with dementia. The guideline content is summarised as officially published, with additional commentary in the final section.

## Key Points

- SIGN168 makes evidence-based recommendations in the diagnosis, care and management of people with dementia and their carers.
- Topics covered are those prioritised by people with lived experience of dementia.
- Summary includes identification and diagnosis of dementia, investigative procedures, postdiagnostic support, non-drug approaches for distress, changing needs, grief and technology.

## Context and scope

November 2023 saw the publication of a new Scottish Intercollegiate Guidelines Network (SIGN) Guideline (SIGN 168) aiming to provide evidence-based recommendations for best practice in the assessment, care and support of adults living with dementia [1]. The increasing prevalence, emerging knowledge around risk factors and recognition of the unequal impacts in terms of risk and experiences of care, all provide a persuasive backdrop for an updated and evidence-informed approach. The guideline follows the recent publication of a Scottish Dementia Strategy ‘Dementia in Scotland: Everyone’s Story’ [2].

Early in development, it was appreciated that a comprehensive review across the dementia journey may not be possible in a single guideline. Although the guideline offers recommendations pertinent to most dementias, it does not address the needs of specific groups such as people with Intellectual and Developmental Disabilities. Topics featured in this guideline are those prioritised by stakeholders, especially people with lived and living experience. To avoid duplication, some topics used evidence-based recommendations already formalised in the National Institute for Health and Care Excellence (NICE) guidelines [3]. Each guideline section contains evidence-based recommendations and good-practice points. Recommendations on areas requiring further research and suggestions for implementation are also included. Table 1 lists the topics covered in this SIGN guidance and highlights areas not covered.

**Table 1:** Summary of major topics covered in SIGN 168 and related topics that are not

Topics covered	Notable related topics not covered, along with rationale
**Identification and diagnosis of dementia** Brief cognitive testing In person and remote testing Informant questionnaires Self-complete questionnaires Discussing a diagnosis Timing of diagnosis	The focus here is on diagnosis in primary care and non-specialist settings. There are no specific recommendations around neuropsychological testing, computerised testing, or assessing for comorbid mood disorder. Experience of diagnosis is included but not experience of screening, this may be due to lack of primary research in this area.
**Further investigative procedures** Amyloid Positron Emission Tomography (aPET) Fluorodeoxyglucose (FDG)PET aPET for differentiating dementias Clinical use considerations of aPET CSF biomarkers CSF for differentiating dementias Clinical use considerations of CSF Diagnosis of specific dementia syndromes Genetic testing	The guidance does not include tests that have clear and relevant recommendations in other guidelines, e.g. Dopamine active transporter (DAT) scanning is covered in NICE guidance. Novel biomarkers not yet used in the clinical routine are not considered, e.g. Tau PET, blood-based biomarkers. Throughout the guideline, the focus is mainly older adult dementia, and investigating young or rapid onset dementia is not covered.
**Postdiagnostic support** Experiences of postdiagnostic support Needs and unmet needs Postdiagnostic support for ethnic minority groups Continuity of care needs and models of delivery Postdiagnostic support in young-onset dementia Postdiagnostic support for young carers	The guideline is designed for the Scottish context, where postdiagnostic support has been established. The background and evidence for postdiagnostic support, and potential content of a postdiagnostic support package are not covered.
**Non-pharmacological approaches for distressed behaviours** Assessment of distress Identifying triggers Interventions for distress and agitation Sleep problems Use of technology for monitoring or support	Not all behavioural and psychological symptoms of dementia are covered, with the focus mostly agitation and aggression. Single component interventions are not comprehensively assessed. This is due to the very large number of non-drug therapies and wide range of presentations of distress. Medications for distress are not covered because NICE guidance [3] still applies. Pain is not covered because management is generally pharmacological and is also covered by NICE guidance [3]. Depression in dementia is covered in NICE guidelines, [3] and is not specifically mentioned in this guidance. Managing delirium in dementia is not covered but the reader is signposted to the SIGN guideline on delirium [4].
**Grief in dementia** Pre-death and post-death grief Grief in people with dementia and carers Assessing and managing grief	Guidance is generally on what clinicians should know, but there is little on what to do or how to do it.
**Changing needs through the disease course** Assessing unmet need Palliative care Anticipatory care planning	There is minimal specific guidance on managing changing needs during transitions in care setting, such as admission to hospital or moving to a care home. The guidance encourages identifying unmet needs and setting person-centred goals at each transition but does not specify how this should be done.

**Figure 1:**
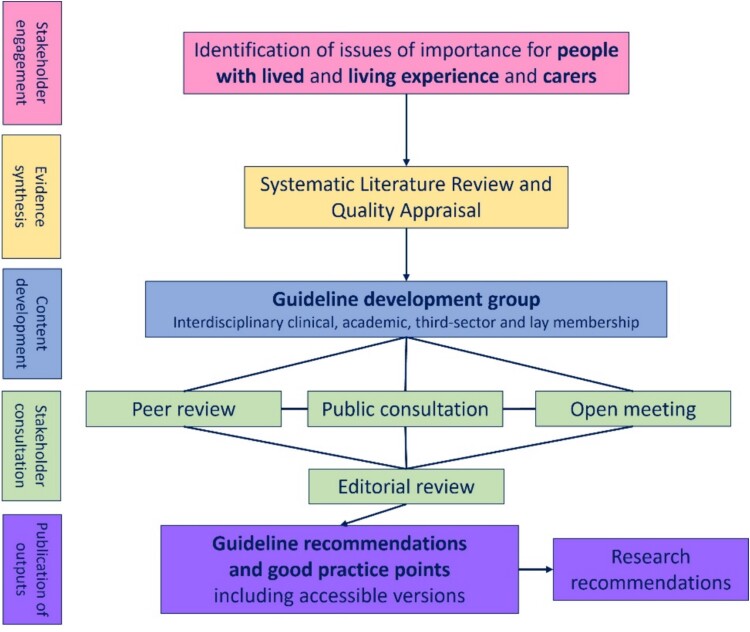
Overview of SIGN 168 guideline production.

Figure 1 summarises key stages in guideline production: initial stakeholder engagement, evidence synthesis, creation of a multidisciplinary writing group, content development and consultation and production of the guideline and other accessible outputs. Full details on the SIGN process are available [5].

This guideline summary includes recommendations related to identification and diagnosis of dementia, investigative procedures, postdiagnostic support, non-pharmacological approaches for distressed behaviours, using technology to support people with dementia, grief and dementia and changing needs of people with dementia. Some content has been shortened for this summary, but the full guideline and references are freely available [6]. This paper summarises the guideline content as published and then offers commentary in the final section. These comments are from the authors of this paper and may not represent the views of SIGN or the Guideline writing group.

## Including those with lived and living experience of dementia

In creating the guidance, SIGN worked in partnership with a variety of professionals involved in dementia care, including those working in primary, community, secondary care, care homes and care at home. In addition to professional stake holders, SIGN included people living with dementia, their families and carers in all stages of guideline production. To ensure that evidence specifically related to this group was included, and to address gaps in academic publications, additional literature searching included reports, websites and resources produced by a range of organisations including: Together In Dementia Everyday (TIDE), Alzheimer Scotland, Age UK, Young Dementia UK, The Alliance, Alzheimer’s Society, Age Scotland, About Dementia and Life Changes Trust. Key themes, information and quotations were extracted and compared to findings raised in consultation with stakeholders. Additionally, the guideline deliberately avoids duplicating guidance in the NICE Guideline [3].

Recognising the importance of experiential evidence for these topics, qualitative and mixed methods studies were included in the evidence synthesis. These publications were appraised using validated tools [7, 8] with quality definitions identified prior to assessment. Qualitative literature summaries and consultation materials were considered throughout the guideline development to ensure that there was a focus on what matters to people with dementia and their families and carers.

To ensure accessibility and utility of the guidance for all stakeholders, SIGN have produced a summary of recommendations with signposting to other forms of support, as well as easy read and audio versions [9].

## Person-centred care

The term person-centred is used in the guideline, although not formally defined. A commonly used definition is included in the NICE Guideline on dementia:

‘The principles of person-centred care underpin good practice in dementia care. . .,. These principles assert as follows:

- the human value of people living with dementia (regardless of age or cognitive impairment) and their families and carers
- the individuality of people living with dementia, and how their personality and life experiences influence their response to dementia
- the importance of the person’s perspective
- the importance of relationships and interactions with others to the person living with dementia, and their potential for promoting well-being.

Finally, the principles emphasise the importance of taking account of the needs of carers (whether they are family and friends or paid care-workers), and supporting and enhancing their input’ [3].

## Identification and diagnosis of dementia

This guideline section focusses on the identification and assessment of a person with suspected dementia and discussion of the diagnosis. Best practice statements emphasise that dementia is a clinical diagnosis, and clinicians should draw upon information from different sources, including history, examination and ideally informant assessment. An important aspect of initial assessment is to exclude reversible causes of cognitive decline.

Based on systematic reviews of accuracy [10, 11], a range of brief cognitive screening tests are recommended for healthcare professionals to consider. No single test is recommended for all settings and the guideline suggests that published estimates of test accuracy should not be the sole criterion for choosing a cognitive test. Other important factors include time required for administration, accessibility, the need for formal training and reliance on written English/literacy skills.

Tests considered suitable for identifying people who would benefit from referral to secondary care are highlighted in Table 2. The guidance states that remote assessment can be used if required, but in-person assessment is preferred, and highlights potential tools to structure the collateral history taking, e.g. the Informant Questionnaire for Cognitive Decline in the Elderly (IQCODE) [12].

**Table 2:** Recommended brief cognitive screening tests to inform referral to secondary care

**Rapid direct tests (taking ≤5 minutes to complete)**: ▪ Clock Drawing Test ▪ General Practitioner Assessment of Cognition (GPCOG) ▪ Mini-Cog ▪ Memory Impairment Screen (MIS) ▪ Six Item Cognitive Impairment Test (6-CIT) ▪ Six-item Screener (SIS) **Extended direct tests (ranging from 10 to 30 minutes to complete):** ▪ Addenbrooke’s Cognitive Examination III (ACE-III) and derivatives ▪ Free-Cog ▪ Mini Mental State Examination (MMSE) ▪ Montreal Cognitive Assessment (MoCA) ▪ Rowland Universal Dementia Assessment Scale (RUDAS) **Self-completion questionnaire:** ▪ The Test Your Memory (TYM) **Informant questionnaires (if a suitable informant is available):** ▪ AD8 ▪ IQCODE **Remote cognitive assessment (where required):** ▪ Telephone Interview for Cognitive Status (TICS) and derivatives ▪ Tele-MMSE ▪ Tele-Free-Cog

### Who should be involved in diagnosing dementia?

**Figure 2:**
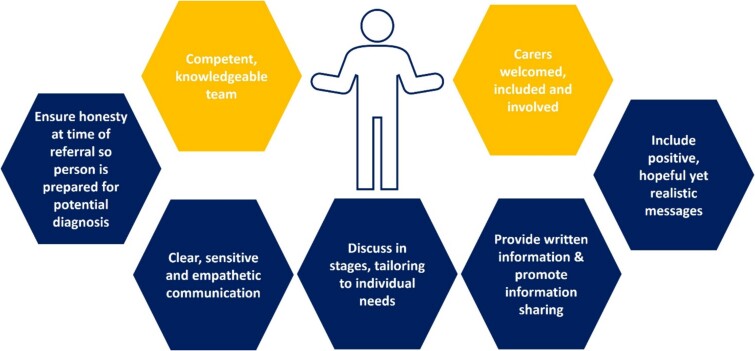
Recommendations to support a positive experience in dementia diagnosis.

Figure 2 provides a graphical summary of what a positive experience of receiving a diagnosis should involve. For making the diagnosis, the guidance recommends that all people with specialist expertise in dementia, including GPs and members of the multidisciplinary team, can have a role. Emphasis is placed not on job title but on competence and knowledge of the condition. Carers are encouraged to be present and actively included in diagnosis discussions, acknowledging the role they play supporting the person with dementia and assisting with communication and recall. Best practice is described as a diagnosis made by someone with suitable training and informed by collateral history, functional assessment and investigations.

There is recognition that people with undiagnosed dementia may have their first contact with healthcare professionals during an acute hospital stay. As a good practice point, the diagnosis of dementia should not be delayed if an expert, supported by multimodal assessment and collateral history, feels confident to identify dementia during a hospital stay.

### Timing of dementia diagnosis

The guideline recommends that early discussion of a diagnosis of dementia is considered; this is to allow timely access to support and services. When sharing a diagnosis, consideration of holding the discussion in stages, allowing time for information to be absorbed and with tailoring around the needs of the person with dementia and their carer(s) is recommended. A person-centred approach to sharing the diagnosis is described as best practice, although it is acknowledged that research to inform this aspect is currently limited.

### Communication about diagnosis

Those making referrals to specialist memory services are recommended to make it clear to the person being referred that they may have dementia, so that they are prepared for a potential diagnosis. Consideration should be given to prediagnostic counselling, and information sharing with people with dementia and their carers should be promoted before, during and after receiving a diagnosis of dementia. This includes information about memory assessments and the medical, interpersonal and behavioural aspects of dementia. Provision of written information is recommended to complement the consultations. The key recommendations around diagnosis are that discussion of a diagnosis should include positive and hopeful, yet realistic, messages. Points to cover include information on prognosis and sources of support, information on wellbeing, and how the person with dementia can continue with their life, maintain their sense of self and accept their identity as someone with dementia. The Healthcare professionals providing the diagnosis should do so in a clear, sensitive and empathetic manner, being aware of differing communication needs and providing opportunity for questions.

## Further investigative procedures

After comprehensive assessment, additional investigations can be considered to exclude other causes of cognitive decline or to support diagnosis of specific dementia subtypes. Echoing previous NICE guidance, the recommendation is that structural brain imaging should be used to rule out reversible causes of cognitive decline and to assist with subtype diagnosis, unless diagnosis and subtype are already clear. Further tests should only be considered if they would change management. The primary new recommendation in this section is that advanced imaging such as amyloid positron emission tomography (PET) should not be routinely used to make a diagnosis.

Accepting that there are situations where imaging or tissue biomarkers may be useful, best practice is to only consider these additional investigations after full clinical assessment and with discussion of potential risks and benefits. There is acknowledgment that evidence around efficacy of these tests is mixed [13]. In terms of choice of test, two recommendations are reproduced directly from NICE guidance [3]:

▪ If the diagnosis is uncertain and Alzheimer’s disease is suspected, consider either:
  - FDG-PET (fluorodeoxyglucose-PET-CT), or perfusion SPECT (single-photon emission CT) if FDG-PET is unavailable or
  - Examining cerebrospinal fluid (CSF) for: either total tau or total tau and phosphorylated-tau 181 and either amyloid beta 1–42 or amyloid beta 1–42 and amyloid beta 1–40.
▪ If a diagnosis cannot be made after one of these tests, consider using the other one.

The potential for a single gene disorder as a cause for dementia in small groups of patients is acknowledged, especially for those with frontotemporal dementia, early-onset Alzheimer’s, Huntington’s chorea or motor neurone disease features. Best practice is described as only considering genetic testing in individuals with age of onset less than 55 years, or with family history of dementia of same type in first or second degree relative. The guideline signposts to national and local genetic testing protocols and resources [14].

## Postdiagnostic support

More than in other sections of the guideline, the recommendations around postdiagnostic support are shaped by the Scottish context [15]. The Scottish standard around postdiagnostic support is that people newly diagnosed with dementia will be offered a minimum of 1 year support, coordinated by a named link worker and based on models developed by Alzheimer Scotland [16, 17]. The Guideline does not specifically endorse this model of postdiagnostic support, and the evidence considered by the guideline writing group suggested benefits and limitations [18, 19]. However, the recommendations that follow are designed to work within this context.

Evaluating evidence around experiences of postdiagnostic services, the guideline recommends that postdiagnostic support should be co-ordinated between services, meets the needs of people with dementia and their carers throughout all stages of dementia, and encourages their engagement with services**.** The key recommendation for this section is that services should offer continuity of care with a single point of contact. Person centred care is emphasised throughout the guideline, and this remains relevant to the post diagnostic support section.

Consideration of carers’ preferences for psychosocial support and education and efforts to address stigma are emphasised. Especially around how the person with dementia and their carer(s) view the illness and options for care, based on their knowledge, experience and cultural beliefs. This is to encourage health-seeking behaviours and uptake of care and services.

**Figure 3:**
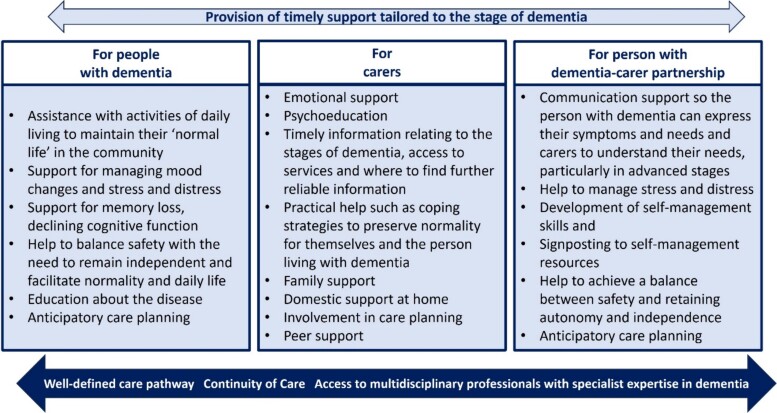
Potential components of postdiagnostic support.

Postdiagnostic support should address the needs of the person with dementia, carers and the person with dementia–carer partnership (i.e. where there is a dyad of a person with dementia and their carer). Recommendations of what postdiagnostic support should include are summarised in Figure 3.

There is a recommendation that support should be flexible, sensitive and suited to the needs of the person with dementia and their carers. This includes tailoring the format of information being provided mindful of literacy, language, additional support needs and cultural sensitivity. The guideline acknowledges the need for a holistic assessment, which includes considering other risk factors and comorbidities.

Professionals are recommended to signpost to both non-statutory organisations (e.g. charities/voluntary organisations) that offer social and emotional support, and to reliable information sources including websites and social media (blogs, online forums).

Similar to sharing diagnosis, the guideline recommends that delivery of postdiagnostic support should not be the exclusive remit of a particular discipline and may be suited to those working in health and social care services. In this context, expertise in dementia is evidenced by recognised national knowledge and skills development underpinned by the Promoting Excellence Framework [20].

A process of education around dementia and its management, including communication skills and person-centred approaches to care, is suggested. Empathetic communication is recommended for all professionals caring for people with dementia, and enhanced communication skills are highlighted to support effective communication for people living with advanced dementia.

Multidisciplinary expertise from old age psychiatry, geriatric medicine and other specialist disciplines related to comorbidities that often accompany dementia are also recognised as being part of postdiagnostic care.

### Groups with specific postdiagnostic support needs

While the guideline emphasises the need for postdiagnostic support to be bespoke to the individual, there is a recognition that certain groups may have specific needs that are not best served by the usual approach.

For those from ethnic minority groups, the guideline recommends provision of culturally sensitive services and information, in the language most suited to the individual and their support network. The guideline highlights concern around the possibility of stigma among minority ethnic populations, associated with reduced recognition of dementia as an illness, and increased likelihood to ascribe symptoms to the ageing process. Professionals should be mindful of such stigma when engaging with these groups.

Recognising the distinctive needs of those receiving a diagnosis of young-onset dementia, the following are recommended:

- immediate emotional support, in the short term, rather than information provision
- age-appropriate support services (e.g. appropriate day services) and programmes, tailored to their individual needs, and support with accessing these
- information on prognosis, services and coping strategies
- support to engage in meaningful activities
- peer support
- support with work and employment
- support with financial problems.

People with young-onset dementia should be offered a key worker or case manager to provide continuity of support and enable the person living with young-onset dementia to understand their condition and actively engage in their care plan and journey. The key worker should act as a co-ordinator of services, organisations and people and be available through a variety of organisations, depending on local infrastructure, such as primary care or the voluntary sector, or within local mental health teams or neurology services.

Greater awareness of young-onset dementia and the needs and experiences of those with a diagnosis and their families, including support with grief, is recommended. Professionals should be aware that denial, refusal to seek help, stigma associated with the disease, and the emotions associated with diagnosis, and sharing this diagnosis with others are major barriers to accessing care in this population.

A whole family approach is recommended for people with young-onset dementia and any young carers. This should include tailored and co-ordinated support and care from all sectors (including adult and child health services, education and voluntary sectors). There should be a focus on alleviating care burden and ensuring the young carer continues with education or work. All should work flexibly and cohesively to support the young carer’s needs, facilitate continuity and look for and address any stigma or bullying.

## Non-pharmacological approaches for distressed behaviours

Distress in this context is defined in an inclusive way, recognising the unique and individual ways in which a person living with dementia may experience it. There is an acknowledgement of the broad range of terminology used in literature and practice, and a recognition that there is no perfect single term to capture this complex concept. Restlessness, pacing, repetitive questioning, agitation, aggression, resisting or not engaging with interventions, apathy and sleep disturbance are all considered potential manifestations of distress, but others are acknowledged.

Distress is also highlighted as an important issue for those experiencing delirium, a diagnosis that is more common in people living with dementia. Evidence-based recommendations around distress and delirium are presented in SIGN 157 (risk reduction and management of delirium) [4].

In the guideline, distress is conceptualised as a potential sign of unmet needs—biological, psychological, social or environmental. For this reason, non-pharmacological approaches are recommended as first line for responding to individuals with distressed behaviours, with a focus on trying to identify unmet needs. The guideline focuses solely on non-pharmacological approaches for distress, as evidence around pharmacological approaches for managing distress is already covered in recent National Institute of Health and Care (NICE) dementia guidelines [3]. The guideline group acknowledges that providing recommendations is complicated by the lack of high-quality evidence assessing models of care used in contemporary clinical practice. This may explain why assessment and management of distressed behaviours varies substantially in practice.

Assessment is identified as a specific area of interest, but no evidence-based recommendations are made. Instead, a good practice recommendation supports the holistic assessment of the individual with distressed behaviour. This can be supported by structured and objective measures to recognise distress, and functional analysis to identify possible triggers.

The key recommendation in the section is that activities tailored to the individual, a focus on preserved capabilities, and considering the person’s previous roles, interests and preferences can be considered for managing distressed behaviours. These should be based on a comprehensive, structured assessment of symptoms. For people living in care homes, a multicomponent programme, which includes support and training for staff, and content tailored to the needs of the person living with dementia, can be considered.

To support carers of people with dementia in managing distress, tailored psychosocial education and skills training can be considered. Suggested components for this training may include the following:

- problem solving
- identifying triggers
- coping strategies for distressed behaviour
- stress reduction
- cognitive restructuring
- communication skills
- crisis management.

Good practice recommendations acknowledge the need for staff training to support management of distressed behaviours and improve the quality of life for people with dementia. This should be part of ongoing multidisciplinary skills development and take place with leadership, infrastructure and resources to enable implementation and engagement.

Among the many manifestations of distress, sleep disturbance was identified as a priority topic. A multicomponent approach with sleep hygiene education, exposure to daylight, exercise and personalised social activities included is suggested.

## Using technology to support people living with dementia

There has been a rapid expansion in technologies to support people living with dementia and their carers. Not all the international evidence was considered relevant, as technological interventions are impacted by the context and setting of health and care delivery. Available studies generally considered short-term interventions and used remote technologies in home settings.

**Figure 4:**
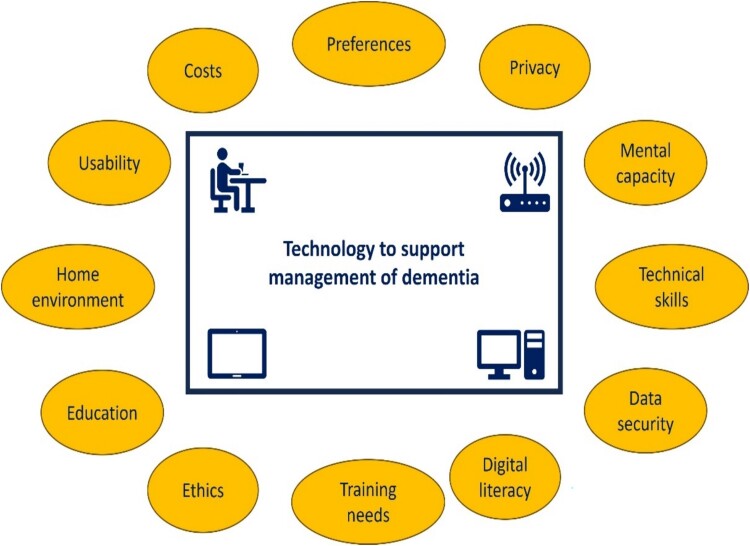
Key considerations when using remote technologies to support person and carers.

Recommendations are made around key considerations when using remote technologies to monitor or support a person with dementia and their carers in a home setting (Figure 4**)**. These include being mindful of preferences which may exist for either face-to-face contact or online consultation. The role of face-to-face contact in enabling professionals to notice changes in the person with dementia is highlighted. Practical, physical and ethical considerations include the skills (technical, digital literacy, mental capacity and competency), training and education needs of the person with dementia and their carer(s); ease of use of technology; physical changes required in the home environment and the cost, data security, ethical (e.g. informed consent) and privacy issues posed by use of technology.

## Grief and dementia

The topic of grief and dementia is explored in detail, recognising it is an area which has been under-recognised, under-researched and in which individuals feel unsupported. It is also an area that has been given limited attention in previous dementia guidelines. The key recommendation in this section serves to raise awareness, stating that healthcare professionals should be aware that carers of people with dementia may experience predeath grief from the point of diagnosis and throughout the stages of dementia. Other recommendations are structured around anticipatory or predeath grief (affecting the person with dementia and/or their carers), grief among people with dementia following bereavement and grief following bereavement in carers including prolonged and complicated grief.

### Anticipatory/predeath grief

The guideline emphasises the grief-like experiences which a person living with dementia may experience. These may be in relation to the progressive loss of personal identity which can result in mourning for their previous self; reflections about their death; loss of their plans for the future; and loss of social roles, relationships and competencies. In much of the evidence considered, it is noted that the perspectives of people with dementia have typically been researched alongside their family members or carers, rather than being considered in their own right.

For carers of people with dementia, there is encouragement for professionals to enquire sensitively about the topic of grief. Holistic assessment of caregivers is recommended to inform management strategies that will enable them to cope with their caring responsibilities and daily tasks. Increasing severity of illness and transitions of care, such as moving-in to long-term care, are associated with worsening of predeath grief experiences among carers. There is also an association between predeath grief in carers and depression, and the guideline highlights the need to avoid misdiagnosis. While depression and predeath grief may co-exist, treatments may not be effective for both aspects, and the distressing and disabling nature of predeath grief should be recognised in terms of the negative impact on individual wellbeing. Similarly, good practice statements explain that grief should not preclude a carer from receiving treatment for depression.

**Figure 5:**
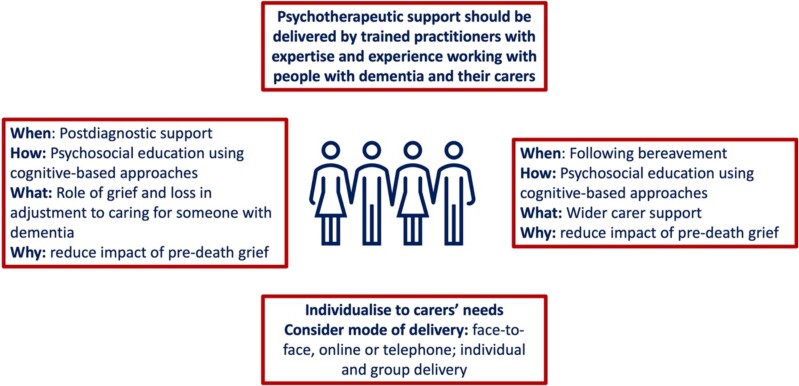
Support and management approaches for carers with pre-death grief Note that the experience of grief can start at diagnosis and so is both part of postdiagnostic support and support following bereavement.

Figure 5 summarises key recommendations in how to support and manage the needs of carers with predeath grief. Health and social care professionals should receive guidance and training on the assessment and support of predeath grief for carers of people with dementia. Approaching assessment, support and management should focus on carers and family units, as well as people with dementia, and could include coping skills for loss and grief.

### Grief following bereavement

The guideline highlights that when a person living with dementia experiences the loss of a spouse or family member, this loss and the associated grief may be re-experienced if the person is unable to retain details of their loss. Additional professional support may be needed to manage grief after bereavement, to process the loss and to manage any associated distress, agitation or confusion. Specific national training exists to enable professionals to deliver such support.

Individuals who have cared for someone living with dementia are at risk of complicated or prolonged grief. The latter is defined as grief persisting for more than 6 months causing significant impairment in functioning. Such reactions are more likely among spouses, if the person with dementia moves into long-term care or among those who have experienced high levels of guilt, depression, a lack of social support and a higher care burden. The guideline suggests a potential role for psychological support where the carer agrees.

## Changing needs of people with dementia

The progressive nature of dementia provides the framing for the final Guideline chapter, which makes recommendations around palliative care approaches and future care planning. Transitions of care, such as hospital discharge or moving-in to long-term care, provide important triggers to review, update or initiate care planning that captures needs and wishes, including care and support plans.

### Palliative and end-of-life care approaches

A key challenge when supporting people with dementia is around definitions of end-of-life, as distinct from the broader framework of palliative and supportive care. Palliative care is conceptualised as on-going from diagnosis to end of life. The evidence considered leads to a key recommendation that existing measures to define end of life in people with dementia, based on cognition and ambulatory function, may be ineffective and may not recognise unmet needs at end of life. This is an important consideration for healthcare professionals who are advised to focus on the needs of the individual rather than defined disease stages. Furthermore, dementia is recognised as a common cause of death, and education should be available for families and carers to understand how this occurs.

Some recommendations are reproduced from the NICE guideline on dementia:

▪ From diagnosis, offer people living with dementia flexible, needs-based palliative care that takes into account how unpredictable dementia progression can be.
▪ For people living with dementia who are approaching the end of life, use an anticipatory healthcare planning process. Involve the person and their family members or carers (as appropriate) as far as possible, and use the principles of best-interest decision-making if the person does not have capacity to make decisions about their care.

### Transitions between care settings

Holistic, person-centred approaches should be considered when people with dementia transition between care settings. This includes identifying unmet needs such as depression, physical decline (evidenced by falls or walking and balance issues) and problems with basic activities of daily living. Having identified unmet needs, there is an opportunity to set person-centred goals and make care plans to address these needs and signpost to or link with appropriate services that offer support. It is recognised that transitions into long-term care often occur when a person is in the more advanced stages of their illness and their involvement in goal-setting may be more challenging. Early discussions are recommended to identify an individual’s expected needs, goals and plans to support their transition when the time arises.

### Advance and anticipatory or future care planning

Here, terminology has shifted from advance care planning, to anticipatory care planning, with its broader focus on living with long-term conditions. There is a national shift toward using future care planning in this context. People living with dementiacan benefit from engaging in care planning. This should be provided early, and on an on-going basis, for the person living with dementia and those people involved in their care. Topics that should be discussed include as follows:

- The benefits of planning ahead.
- Lasting power of attorney (for health and welfare decisions and property and financial affairs decisions).
- An advance statement about their wishes, preferences, beliefs and values regarding their future care.
- Advance decisions to refuse treatment.
- Their preferences for place of care and place of death.

It is important that people are aware that information and decisions can be reviewed and changed and that opportunities are provided to enable this.

It is recommended that anticipatory care planning discussions should: be tailored to the needs, readiness to engage and capacity of the person with dementia; consider the needs of family and carers and consider triggers for discussions, such as diagnosis, change or decline in health status or change in place of residence. Awareness among people with dementia and their family and carers should be raised around the progressive nature of the condition and around what to expect at different stages of the illness. Receptivity to anticipatory care planning discussions is increased when those involved have insight into the progressive and terminal nature of dementia. Anyone should have the opportunity to initiate and be involved in anticipatory care planning discussions. Successful discussions should be based on trusting relationships. If discussion is not initiated by the person with dementia themselves, healthcare professionals should proactively initiate or enable person-centred conversations as soon as possible. Anticipatory care planning can take place in any care setting, including the family home, primary care, hospital or care home.

The key recommendation in this section is that anticipatory care planning may require a series of conversations over time to allow clarification, reflection and updates to the plan to reflect any changing needs. Early discussions are beneficial, as the capacity of the person with dementia will diminish with disease progression. This needs to be underpinned by education and training for professionals on communication skills, anticipatory care planning, the dementia disease trajectory, treatment and care options, and palliative care.

## Research recommendations

Despite the focus of topics on the experiences of living with dementia, there remained a substantial lack of published research that includes the views of people living with dementia. This prompts a call to action for the normalisation and prioritisation of such evidence, to help understand the experiences of living with dementia. Twenty-eight specific topic areas specific to guideline topics included in this summary were identified **(**Table 3**).**

**Table 3:** Research recommendations

*Dementia diagnosis and discussion of diagnosis* ▪ Perceptions of people with dementia on timing and processes of dementia diagnosis discussions. ▪ Country-specific studies in relation to dementia diagnosis. ▪ Timing of diagnosis and role of prediagnostic counselling. ▪ Where the dementia diagnosis discussion should take place. ▪ The role of the MDT in dementia diagnosis. ▪ The impact of training in communication about disclosure and methods of communication on people with dementia and their carers. *Brief screening tools* ▪ More research on telephone and video-based screening tools. These studies should assess accuracy, feasibility and acceptability in real-world populations. ▪ Research on the accuracy, feasibility and acceptability of new screening tests, such as Free-Cog and Oxford Cognitive Screen. *Remote assessment* ▪ The feasibility, acceptability and supportiveness of remote assessment in the diagnosis of dementia. *Fluid biomarkers* ▪ Evaluation of emerging tau PET tracers against alternatives. ▪ Long-term follow-up of people with different clinical diagnoses and positive or negative CSF biomarkers for Aβ42, T-tau and P-tau, including eventual postmortem examination validation. More data are required on biomarkers’ diagnostic value in older people who are more likely to have Alzheimer’s disease copathology. *Postdiagnostic support* ▪ Postdiagnostic support that relates to the stages of dementia and specific populations. ▪ Research into the experiences of postdiagnostic support in minority groups, those with protected characteristics, those with young-onset dementia and young carers. ▪ Postdiagnostic support specific to people with advanced dementia. ▪ How to achieve person-centred care. ▪ Models of care and the various multidisciplinary contributions. *Distressed behaviours* ▪ Research describing the assessment of, and process for arriving at decisions on person-centred interventions, for individuals who experience distress. ▪ Non-pharmacological therapies and the effectiveness of such therapies for distressed behaviours. ▪ Understanding how carers communicate and interact with a person when they are distressed, including communication styles, methods and de-escalation. *Grief and dementia* ▪ Acceptability of grief interventions for men and minority ethnic groups. ▪ People with dementia’s experience of pre-death grief and bereavement and how to support them. ▪ How people with mild to moderate dementia experience grief and how to help them retain and process the pertinent information about their loss. ▪ Interventions to support carers with grief before and after death. ▪ Healthcare professionals’ experiences around grief in dementia before and after death. *Changing needs in people with dementia* ▪ Assessment of approaches to address changing or unmet needs in people with moderate, advanced or severe dementia. ▪ Describing the unmet needs of people with young-onset dementia. ▪ An approach to regular assessment of comorbidities and cardiovascular risk factors, and the implications of holistic or integrated care assessment. *Anticipatory care planning* ▪ The utility of ACP during the earlier stages of dementia.

## Commentary

In a time of substantial change in dementia theory and practice, a new guideline on dementia is welcome. Given the complexity of dementia care and the increasing published science, it would not have been possible to deliver timely guidance that offered comprehensive recommendations on all aspects of dementia. It seems appropriate that SIGN chose to focus on key topics, but in an exercise that has to prioritise content, there will also be important topics that do not make the final selection. With the SIGN guideline, there are some fundamental aspects of dementia that were not included, e.g. prevention and brain health. Equally within the chosen topic themes, not all aspects were covered, e.g. the review of interventions was limited to treating the distress syndrome and did not consider interventions to improve overall quality of life, reduce cognitive decline or manage comorbidities. For this reason, some ‘hot topics’ in dementia such as exercise and disease modifying drugs receive no attention. This is not a criticism, and SIGN are to be commended for presenting a clear rationale and transparent process of prioritisation that involved scoping evidence, describing variation in practice [21] and seeking feedback from stakeholders including people living with dementia.

Some of the topics chosen for the SIGN guideline were not suited to the traditional guideline format of meta-analysis and subsequent recommendation. SIGN made use of a variety of evidence synthesis techniques including overview, qualitative and test accuracy meta-analyses [22]. Even with this, there are many areas where an evidence-based recommendation would be difficult to achieve, explaining the large number of best practice points in this guideline. Hopefully, clinicians will appreciate this pragmatism.

In the sections on testing, the evidence is presented in a balanced way considering all the options and describing advantages and limitations of differing approaches. It could be argued that busy clinicians and policy makers would have preferred a recommendation around a single preferred test for cognitive assessment. New techniques in evidence synthesis would allow for a network of direct and indirect comparisons of differing tests and could rank their accuracy, suggesting a favoured test [22]. However, a one size fits all recommendation may not be suited to the differing contexts in which assessment is performed, and the SIGN approach of offering options for testing may ultimately prove to be the most appropriate.

One of the most rapidly evolving aspects of dementia care is around biomarkers. With new developments in blood-based biomarkers [23], there is a danger that the evidence-based recommendations given here, and indeed in any contemporary guideline, may soon be outdated. SIGN differs from some other guidelines in that it fully considers the implementation requirements of the recommendations made. Given the limited UK access to technologies such as amyloid PET imaging, the recommendation that advanced imaging should not (yet) be routine seems appropriate locally but may not be relevant to other healthcare systems.

Although the anticipation with any SIGN guideline is that the guidance will be useful to all clinicians, the primary evidence users are the Scottish Health and Care systems. For dementia, more so than in other diseases, the guidance given was adapted to suit the local context. For example, the section on postdiagnostic support recognised that it is already a national standard to provide a year of a postdiagnostic support package. This explains why the focus of recommendations is more around the delivery of support, rather than asking whether the approach works or the optimal intervention content. NICE guidance does not use the terminology of postdiagnostic support instead referencing the need for review after diagnosis and care coordination, with a resource coauthored with the Social Care Institute for Excellence for people with dementia and their carers [24].

Devoting an entire chapter of the guideline to non-pharmacological treatment of distress is welcome as these approaches are notoriously under-used in many areas of practice, including in dementia [25]. Despite this, clinicians might be left frustrated at the lack of clearer guidance on individual therapies and specific instructions on what they should actually do when faced with a person with dementia in distress. This is partly down to a limited base but mostly reflects the diversity of personalities, preferences and needs of those living with dementia, such that only a person-centred, multi-component assessment and individually tailored interventions could be recommended for all. In this section, and in other sections throughout the guideline, evidence-based recommendations are made, but the specifics of how these should be implemented are often left to practitioners. This offers a degree of flexibility for clinicians and teams, but for areas such as management of distress, where there are so many theories and models, more operationalised guidance may have been preferred.

‘Distress’ can manifest itself in many different ways, but the guideline only considered evidence for reducing distress manifesting as aggression, agitation or sleep disturbance. It is therefore possible that useful interventions for other symptoms may have been missed. Moreover, identification of possible triggers for distress should not be forgotten and may require skilled assessment, given that potential causes are so numerous, from every-day stimuli (or the lack of them) in the environment to unarticulated symptoms such as pain, to undiagnosed medical conditions. This lack of specificity represents a major challenge to the Guideline’s goal of improving dementia care. Since an important way of quality assuring healthcare is to demonstrate compliance with the highest standards of care, how can individual clinicians and healthcare organisations demonstrate an adequate standard against a recommendation that essentially states each person needs their own bespoke plan? How can we know the plan is really the right one for that person? Despite identifying 45 tools to assess distress in dementia, the guideline did not find sufficient evidence to recommend the use of any particular one. There will therefore be challenges in measuring the impact of the guideline and quality-assuring appropriate use of non-pharmacological therapies for distress.

Although not explicitly stated, the section on use of technology appears restricted to technological applications used as a direct replacement for face-to-face care. There is no consideration of use of technology for other applications, such as adaptive or assistive technology, or tele-care, although a recent randomised controlled trial found these were ineffective anyway [26].

Arguably one of the most surprising and innovative aspects of the guideline is the lengthy section on managing grief in dementia, introducing terms such as ‘anticipatory grief’ and ‘pre-death grief’. These are concepts that many clinicians may not be aware of but were highlighted by people with lived experience as important to them. While acknowledging grief is normal, the guideline argues for proactive assessment and counselling even where grief is not complicated by a diagnosable abnormal grief reaction or depression. Moreover, one of the recommendations is that:

‘healthcare professionals should offer a holistic assessment of carers that includes pre-death grief, with consideration of appropriate management and interventional strategies.’ [6].

This clearly goes beyond taking a good collateral and social history and raises some issues around responsibilities and duty of care that goes beyond the referred patient to other individuals within the carer team. Nevertheless, the guideline cites evidence from small trials and qualitative studies that such a strategy would improve experiences of grief if done correctly [27, 28]. It is unclear if this will be achievable at scale or indeed if it is cost-efficient especially given the acknowledgement in the guideline of evidence that it is possible to worsen grief with such interventions [29]. Some recommendations involve clinicians ‘being aware’ of grief and the problems it causes, but the guideline does not present any evidence that improving awareness among professionals lessens grief or what exactly clinicians can do to help beyond enquiring about grief and referring onwards to counselling services that may not exist in many areas. Nevertheless, publication of the guideline may encourage the development of relevant knowledge and skills among health and social care professionals, enabling more effective postdiagnostic support and a better experience of dementia. It also provides an opportunity for practice-based research to develop evidence around utility and cost-effectiveness.

There are well-recognised tensions around both improving recognition and support for the progressive and fatal condition of dementia while also addressing and lessening stigma through encouraging people to live well with dementia. The latter is a pervasive concern for people living with dementia and their experiences within wider society including health and social care professionals and services. The guideline’s focus on palliative care and anticipatory planning from the point of diagnosis is therefore laudable, as is the explicit recommendation that family and carers with dementia be helped to understand that dementia can be the cause of death. Planning for future events can be challenging in any setting and condition, but the opportunity to do so is generally welcomed and taken up even in the frailest of individuals [30].

## Conclusions

This new SIGN guideline offers a contemporary evidence-informed approach to the assessment, diagnosis, care and support for people with dementia and their carers and the evidence applicable after initial diagnosis to support people living with dementia and those who are important to them. Core topics for clinical practice, such as supporting those with distressed behaviours, are included alongside less recognised but nonetheless important areas such as grief in dementia. Recommendations are practice focused, reflecting the available evidence and highlighting key gaps. Closing these gaps through practice-focused research is essential to improve the care and experiences of those living with dementia in the future. While the guidance represents current best practice, it is likely that revisions will be required to keep pace with new developments.
